# LRRK2 and Proteostasis in Parkinson’s Disease

**DOI:** 10.3390/ijms23126808

**Published:** 2022-06-18

**Authors:** María Dolores Pérez-Carrión, Inmaculada Posadas, Javier Solera, Valentín Ceña

**Affiliations:** 1 Unidad Asociada Neurodeath, Universidad de Castilla-La Mancha, 02006 Albacete, Spain; mariad.perez@uclm.es (M.D.P.-C.); inmaculada.posadas@uclm.es (I.P.); 2 Centro de Investigación Biomédica en Red Sobre Enfermedades Neurodegenerativas, Consorcio CIBER, Instituto de Salud Carlos III, 28029 Madrid, Spain; 3 Servicio de Medicina Interna, Complejo Hospitalario Universitario de Albacete, 02006 Albacete, Spain; solera53@gmail.com; 4 Facultad de Medicina de Albacete, Universidad de Castilla-La Mancha, 02006 Albacete, Spain

**Keywords:** Parkinson’s disease, LRRK2, proteostasis, chaperones, autophagy, LRRK2 silencing, α-synuclein

## Abstract

Parkinson’s disease is a neurodegenerative condition initially characterized by the presence of tremor, muscle stiffness and impaired balance, with the deposition of insoluble protein aggregates in Lewy’s Bodies the histopathological hallmark of the disease. Although different gene variants are linked to Parkinson disease, mutations in the Leucine-Rich Repeat Kinase 2 (LRRK2) gene are one of the most frequent causes of Parkinson’s disease related to genetic mutations. LRRK2 toxicity has been mainly explained by an increase in kinase activity, but alternative mechanisms have emerged as underlying causes for Parkinson’s disease, such as the imbalance in LRRK2 homeostasis and the involvement of LRRK2 in aggregation and spreading of α-synuclein toxicity. In this review, we recapitulate the main LRRK2 pathological mutations that contribute to Parkinson’s disease and the different cellular and therapeutic strategies devised to correct LRRK2 homeostasis. In this review, we describe the main cellular control mechanisms that regulate LRRK2 folding and aggregation, such as the chaperone network and the protein-clearing pathways such as the ubiquitin–proteasome system and the autophagic-lysosomal pathway. We will also address the more relevant strategies to modulate neurodegeneration in Parkinson’s disease through the regulation of LRRK2, using small molecules or LRRK2 silencing.

## 1. Introduction

Parkinson’s disease (PD) is a neurodegenerative condition that progresses with age and causes both mental and physical disability [1,2]. It has been classified as the second most common disorder derived from neuronal degeneration and its incidence will increase during the coming years due to population ageing and lifestyle. It is expected that it will affect between 12 and 17 million people worldwide in 2040 [3]. After a prodromal period, PD is clearly recognized by several movement-associated symptoms such as tremor, bradykinesia and postural imbalance. Motor symptoms aggravate with the progression of the disease and patients also develop non-motor symptoms, including cognitive impairment, sleep disorders and gastrointestinal or olfactory disturbances [4]. Clinical symptoms are mostly derived from the reduction of dopamine levels in the striatum, due to dopaminergic neuronal loss in the substantia nigra pars compacta (SNpc), and the imbalance between the dopaminergic and cholinergic activity. Like in other neurodegenerative disorders, the presence of insoluble aggregates, composed by toxic misfolded proteins, is the hallmark of the disease. In particular, the deposition of α-synuclein (α-syn) in cytoplasmatic insoluble inclusions constitutes the characteristic Lewy’s Bodies and Lewy’s Neurites, the histopathological hallmark of the disease [5].

PD affects both men and women, but there are some gender differences in prevalence, progression of the disease, clinical manifestations and response to pharmacological therapies. On the one hand, men have a higher risk factor for developing PD than women. The incidence of the disease is higher in men than women and the age of onset is lower, especially for the range from 50 to 59 years old [6]. As consequence, women are less represented in PD clinical trials compared to men [7]. However, men live more years following the diagnosis of the disease while PD progression is faster in women [8]. Moreover, women PD patients present more side effects associated with pharmacological therapies. Thus, the risk of developing dyskinesia and the classical on-off episodes is higher in women [9,10]. Additionally, there are also some gender differences according to clinical symptoms. Although cognitive decline is more prevalent in men, depression is more frequent in women [11,12].

Etiologically, around 90% of conditions are sporadic forms and only a small proportion of PD cases are related to gene mutations, with a familial origin maintained over generations. In addition, the exposure to specific environmental substances in combination with specific pathogenic variants is also considered a likely cause of dopaminergic neurodegeneration [13,14].

Different gene mutations, in at least 13 different genes, have been identified as PD-genetic causes [15,16], with single nucleotide polymorphisms (SNPs) in the Leucine-Rich Repeat Kinase 2 (LRRK2) gene one of the most frequent. LRRK2 mutations are related to PD with autosomal dominant inheritance and constitute a major genetic risk factor for idiopathic PD (iPD) [17,18,19,20]. Progressive neuronal dysfunction mediated by mutated LRRK2 has been linked to changes in enzymatic activity, aberrant protein-folding and protein aggregation [21].

Proteostasis plays a key role for the maintenance of cell viability by regulating protein synthesis and degradation. The imbalance in proteostasis leads to aberrant protein-folding and the deposition of harmful aggregates in some neurodegenerative disorders, also known as aggregopathies. The most common protein aggregates include amyloid-beta peptide for Alzheimer’s disease, Huntingtin protein for Huntington’s disease, and α-syn for PD [22].

In this review, we will describe the relevance of LRRK2 homeostasis imbalance as an underlying cause for PD. We will also discuss the most important cellular mechanisms involved in preserving LRRK2 homeostasis, and the main pharmacological and genetic strategies to modulate neurodegeneration produced by LRRK2 mutations.

## 2. LRRK2 Structure and Functions

**Figure 1:**
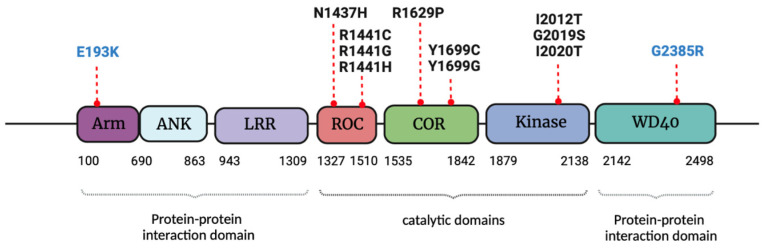
LRRK2 structure. LRRK2 contains seven structural domains, known as armadillo domain (Arm), ankyrin domain (ANK), leucine-rich repeat domain (LRR), ROC domain (Ras of Complex), COR domain (C-terminal of ROC), kinase domain (kinase) and WD40 repeat domain (WD40). PD associated-LRRK2 mutations and risk factors are indicated with a red line above the specific structural domain.

LRRK2 is a large protein of 286 kDa composed by 2527 amino acids, which are distributed in seven structural domains, defined as armadillo domain (Arm), ankyrin domain (ANK), leucine-rich repeat domain (LRR), ROC domain (Ras of Complex), COR domain (C-terminal of ROC), kinase domain (kinase) and WD40 repeat domain (WD40). Functional studies have revealed that LRRK2 is a complex protein with a dual enzymatic activity, as kinase and GTPase, associated with the central catalytic core of the protein, composed by the ROC, COR and kinase domains [23] (Figure 1). LRRK2 kinase domain phosphorylates serine and/or threonine residues in different well-characterized substrates, including α-syn, β-tubulin [24], endofilin A1 [25], synapsin I [26], N-ethylmaleimide-sensitive factor (NSF) [27] and several members of Rab family [28], even LRRK2 itself, through autophosphorylation of serine 1292 [29], threonine 1491 and threonine 2483 [30,31] among other residues [32]. On the other hand, LRRK2 GTPase activity, which is critical for kinase activity regulation, is controlled by the ROC-COR tandem through a GTP binding site [33,34]. The characterization of LRRK2 architecture has also pointed out the relevance of terminal domains for LRRK2 functions. The ARM, ANK and LRR domains, located at the N-terminal region, as well as the WD40 domain, at the C-terminal part of the protein constitute the assembly points for protein–protein interactions [35,36] (Figure 1). Therefore, LRRK2 is considered a scaffolding protein with the ability to regulate organelle transport [37], with the homodimer of LRRK2 the active form [38,39,40]. The LRRK2 signaling network in PD is complex [41] and arises from its interaction with several membrane proteins of different cellular organelles, including synaptic vesicles [42,43], cytoskeleton [24,44], endo-lysosomal structures [45,46] or mitochondria [47,48].

## 3. LRRK2 Pathological Mutations, Gender Influence and Molecular Mechanisms Linked to Parkinson’s Disease

The functional and binding properties of LRRK2 can be modified by different mutations along LRRK2 structure. Currently, at least, ten pathological variants within the ROC (N1437H, R1441C/G/H), COR (R1629P, Y1699C/G) and kinase (I2012T, G2019S, I2020T) regions have been confirmed as dominant familial genetic causes of PD [49] (Figure 1). The G2019S mutation is the most extensive in idiopathic PD patients (iPD) [50] and familial inheritance cases [51,52,53], with variable penetrance among populations. Apart from genetics, gender influence has been analyzed in iPD patients and G2019S mutation carriers. Idiopathic patients showed more severe clinical features than G2019S mutation carriers but there were also some differences according to gender. Although iPD men reported severe motor symptoms and inability to perform daily tasks more frequently, women suffered more non-motor symptoms and more side effects from pharmacological therapy [54]. Moreover, a higher prevalence of PD in female G2019S mutation carriers than in men with the same mutation [55] has been described. The main mechanism involved in the cellular toxicity of G2019S variant is an increase in kinase activity [56,57,58,59]. A recent study suggests the stabilization of the kinase domain of this mutant in a conformation that determines a hyperactive state for the full-length protein [60]. Moreover, most of PD-linked LRRK2 variants share the aberrant hyper-kinase and GTPase activity as the main pathological mechanisms involved in neurotoxicity [61,62]. In addition, mutations in the catalytic core of the protein can determine LRRK2 protein destabilization, abnormal folding and turnover [63,64], and produce protein aggregation and the formation of cytoplasmatic inclusion bodies [65]. On the other hand, the relevance of LRRK2 terminal domains in the pathogenesis of PD has been shown by the identification and characterization of the G2385R and the E193K mutations, in the WD40 and the ARM domain, respectively (Figure 1). The pathological variant G2385R is considered a risk factor for sporadic PD in Asian individuals from China, Korea and Japan [66,67]. It has been shown that gender influence has no effect in the prevalence of G2385R-associated PD [55]. However, a recent study has demonstrated that men present a lower risk of cognitive impairment while women are less prone to suffer autonomic dysfunction [68]. G2385R mutation is located at the C-terminal part of the protein and modifies the biochemical and structural properties of LRRK2. In particular, G2385R mutation alters LRRK2 dimerization [69] and reinforces or hampers its interaction with other proteins in different cell lines. For example, G2385R variant enhances binding affinity of LRRK2 to Hsp90 and Cdc37 proteins in HEK-293FT cells [70], while overexpression of the G2385R mutant in the N2a cell line reduces LRRK2 interaction with different proteins such as synapsin I, β-actin, α-tubulin, and 14-3-3 [71]. Interestingly, mutations around the 2385 position determine different biochemical and functional properties for LRRK2 in different species, which could make the characterization of molecular mechanisms that cause PD in in vitro models harder [72]. The N-terminal domain of LRRK2 also acts as a scaffold domain and participates in the protein aggregation phenomenon [73]. The pathological variant E193K inside the Arm region interferes with LRRK2 protein-folding and the supramolecular LRRK2 organization [74]. In summary, although LRRK2 toxicity has been mainly associated with hyper-kinase activity, this feature is not present in all LRRK2 pathological variants. Instead, alternative mechanisms, such as the loss of LRRK2 stability and correct folding as well as the altered ability to bind with different interacting proteins, could be involved in the pathological mechanisms that trigger PD.

## 4. LRRK2 Homeostasis and Quality-Control Mechanisms

Proteostasis or maintenance of protein homeostasis involves the correct biogenesis, folding, and conformation of proteins, its proper cellular trafficking and localization, as well as its suitable degradation within and outside the cells, to guarantee their right functionality. The dysregulation of any of these processes, either protein synthesis, folding or elimination contributes to neuronal degeneration and ageing [75]. There are different cellular mechanisms that deal with the imbalance of protein homeostasis in PD including stabilization and refolding of target proteins by the chaperone system [76,77], degradation of misfolded or aggregated proteins through the ubiquitin–proteasome system (UPS), and the autophagic-lysosomal pathway (ALP) [78,79]. If these strategies fail or are insufficient to restore protein balance, dangerous insoluble proteins accumulate into intracellular deposits named aggresomes.

### 4.1. Chaperone System: Refolding and Degradation of LRRK2 through the Ubiquitin–Proteasome System

There are several approaches to restore protein homeostasis in the CNS, but chaperones are recognized as an essential regulatory element of the proteostasis machinery, either under normal conditions or stressful situations [80]. The chaperone network is composed of highly conserved and ubiquitously expressed proteins whose main functions include ensuring correct protein-folding, after de novo synthesis or denaturation, and the stabilization of target proteins by acquiring their native stable conformation. Moreover, chaperones control the intracellular transport of proteins to locations where they are functional and promote the assembling of protein complexes [81,82,83,84]. Although there are more than 100 chaperones already characterized, the heat shock proteins (Hsp) group constitutes the most important one. Hsp comprises the families Hsp40, Hsp60, Hsp70, Hsp90, Hsp100, and small Hsp (sHsp). These proteins perform different functions to regulate protein homeostasis. For example, some of them, such as Hsp70, are responsible for stabilizing unfolded proteins by promoting native refolding, while other chaperones, such as Hsp110 or Hsp104, produce protein disaggregation and refolding and even a slow aggregation process [85,86,87,88].

Chaperones also prevent protein aggregation by increasing protein clearance by UPS and ALP. On the one hand, misfolded defective soluble proteins that are not correctly folded are selected for UPS elimination [89]. In a first step of the pathway, the chaperone Hsp70 [90] and the ubiquitin ligases E1, E2 and E3 tag client proteins by the addition of ubiquitin motifs on lysine residues [91], and then ubiquitinated proteins are identified by the 19S proteasomal subunit to eliminate ubiquitin chains, by the specific deubiquitinating enzymes (DUB): USP14, UCH37 and RPN11 [92]. Finally, the specific protein is unfolded and destroyed by the proteolytic 20S subunit in small peptides.

**Figure 2:**
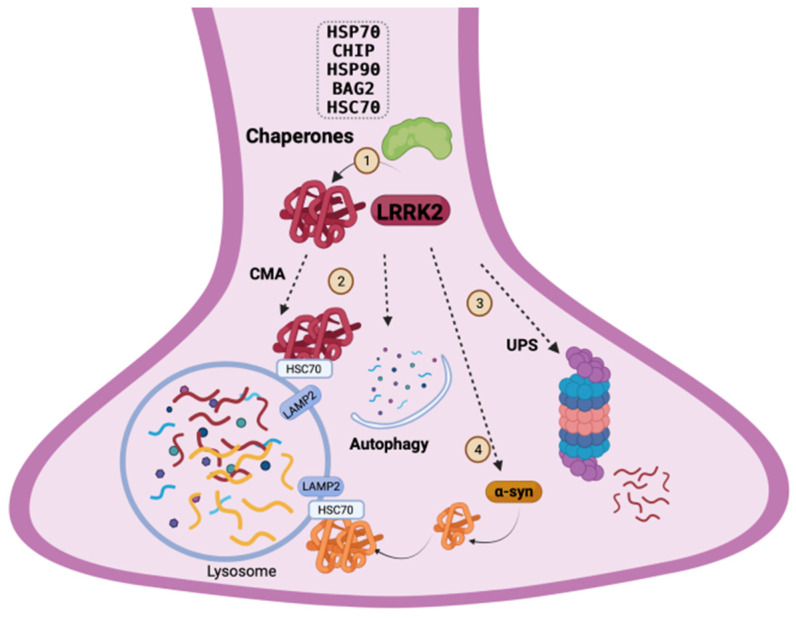
LRRK2 homeostasis and quality-control mechanisms. There are several approaches to control LRRK2 homeostasis: chaperone system (1), chaperone-mediated autophagy (CMA) and macroautophagy (2) and the ubiquitin–proteasome system (UBP) (3). LRRK2 dysregulation contributes to α-synuclein (α-syn) aggregation (4), which is also recognized as CMA substrate.

It is important to highlight the crucial stabilizing role of the chaperone system under physiological conditions, where LRRK2 wild-type is expressed, and in pathological situations derived from point mutations along the LRRK2 structure. Most of the LRRK2 variants share defects on structural conformation and therefore strategies oriented to correct aberrant LRRK2 folding or aggregation are an appropriate alternative to restore LRRK2 homeostasis. Based on chaperone function, Hsp70 overexpression decreases LRRK2 aggregation, without modifying soluble protein levels, suggesting that Hsp70 could control LRRK2 pathological accumulation [93]. The C-terminus of Hsp70-interacting protein (CHIP) is a chaperone with E3 ubiquitin ligase activity. CHIP expression plays a key role in LRRK2 folding, accumulation and toxicity. CHIP binds to LRRK2 and ubiquitinates it to regulate LRRK2 protein-folding and protein levels through proteasomal-dependent degradation of wild-type and familial mutated forms [94,95]. The variant G2385R LRRK2 shows an increased protein turnover because of the higher affinity for proteins that control proteasomal degradation, such as Hsp70 and CHIP [96]. Additionally, different studies have identified the chaperone Hsp90 among the proteins that interact with LRRK2 [97,98]. Hsp90 is essential for controlling LRRK2 stability and steady-state levels of both wild-type and G2019S mutant. Disruption of Hsp90 activity promotes LRRK2 G2019S proteasomal degradation, reducing LRRK2 accumulation and neuronal toxicity derived from the hyperactivity of G2019S mutation [94,95,99]. Moreover, the regulatory function of Hsp90 to manage LRRK2 stability has been also demonstrated for the G2385R variant. The inhibition of Hsp90 leads to the destabilization of the complex and promotes intracellular degradation of the G2385R mutant [96]. However, this mutation shows a different binding affinity for the chaperones Hsp90, Hsc70, and CHIP in several species. Both human and mouse G2385R LRRK2 variants interact with Hsp90, Hsc70, and CHIP, but the mouse mutation binds strongly to these proteins, which could suggest altered LRRK2 folding and stability in this specie [72]. Apart from the classical Hsps, the chaperone complex formed by BAG2 and HSC70 binds to LRRK2 and controls its localization in *C. elegans* [100]. In summary, the regulating role of molecular chaperones in LRRK2 homeostasis seems to be fundamental to keep the right balance between LRRK2 folding and degradation by UPS (Figure 2).

### 4.2. LRRK2 and the Autophagic-Lysosomal Pathway

Although the proteasomal system is an efficient quality-control mechanism for maintaining proteostasis in most situations, there are some limitations that require additional control systems to avoid neuronal degeneration. For example, large aggregates that are not able to access the proteasomal catalytic core by steric hindrance or situations with deficient proteasomal function require alternative mechanisms such as the autophagic-lysosomal pathway (ALP) to guarantee neuronal homeostasis [101].

ALP is an essential quality-control mechanism for the clearance of dysfunctional organelles and long-lived molecules to ensure the renewal of cellular components. It is a complex and tightly regulated catabolic pathway, whose last step involves the lysosomal degradation of intracellular material. ALP is essential to control protein homeostasis in the CNS [102]. Suitable autophagic activity is especially relevant for neuronal protein homeostasis, because neurons are postmitotic cells that are not able to decrease intracellular toxic content by cell division [103]. The reduction of autophagic activity is one of the major causes for generating aberrant protein clearance, and contributes to continuous accumulation of dangerous misfolded proteins into cytoplasmatic protein aggregates [104,105], which are a common pathological feature of the neurodegenerative disorders classified as proteinopathies, such as PD. Moreover, autophagic efficiency declines with age [106], which could facilitate the accumulation of proteins in the form of aggregates and potentiate the spread and progression of PD [107]. For this reason, the stimulation of autophagy has been proposed as one of the main therapeutic strategies to reduce insoluble intraneuronal inclusions in PD [108].

ALP can be classified in three different ways according to the initial step of the pathway: microautophagy, chaperone-mediated autophagy (CMA), and macroautophagy [109]. Microautophagy is the less explored autophagic process, where cytoplasmatic material is directly engulfed and digested by the lysosomes [110]. A specific version of microautophagy that requires late endosomes, which are responsible for sequestering and degrading cytosolic proteins in multivesicular bodies (MVB) has been also described [111]. However, the relationship between LRRK2 and microautophagy has been little explored until now. On the other hand, CMA is an autophagic pathway that performs lysosomal degradation of cytosolic proteins that contain the common recognition motif Lys-Phe-Glu-Arg-Gln (KFERQ) [112]. The cytosolic chaperone heat shock cognate 70 (Hsc70) drives the substrate translocation to the lysosome through the interaction with the transmembrane receptor lysosome-associated membrane protein type 2A (LAMP2A) [113], which multimerize to internalize the substrates into the lysosomes [114]. The relevance of CMA has been demonstrated in PD [115]. LRRK2 shows eight pentapeptide motifs and it is a classical substrate for CMA degradation (Figure 2). LRRK2 wild-type levels increase after LAMP2 silencing [116]. Moreover, the G2019S LRRK2 variant and high levels of LRRK2 wild-type interfere with LAMP2A dynamics, which slow CMA activity and decreases degradation and clearance not only of LRRK2 but of other CMA substrates such as α-syn, whose detrimental accumulation potentiates neuronal toxicity [117]. Similar results were confirmed for LRRK2 R1441G knock-in mice, where LRRK2 mutant altered CMA, decreasing α-syn oligomers clearance [118]. These findings highlight that LRRK2 mutants and LRRK2 wild-type overload disrupts protein homeostasis through CMA.

The best characterized autophagic route is macroautophagy, also referred to as autophagy henceforth. It is mainly characterized by the formation of exclusive LC3-BII tagged-double-membrane vesicles called autophagosomes that finally deliver sequestered cytosolic cargo to the lysosomes. The multi-step process involves the generation of an isolated membrane or phagophore that progressively elongates to trap cytosolic material in a non-selective way (bulk macroautophagy) or after specific recognition and label of intracellular waste (selective or canonical macroautophagy) [119,120]. Based on the nature of intracellular cargo sequestered in the autophagosome, autophagy can be classified in mitophagy (selective degradation of mitochondria) [121], pexophagy (specific degradation of peroxisomes) [122], and the fashionable lipophagy, lysophagy, reticulophagy, nucleophagy or aggrephagy [123]. Defects on these specialized autophagic alternatives have been related to PD [124]. The involvement of the LRRK2 native protein, as well as the contribution of LRRK2 pathogenic mutations in the control of the autophagic pathway, has been extensively studied in several cellular models [125,126,127,128,129,130], animal models, such as Drosophila [131] or mice [132,133,134] and human tissue [135]. Despite the strong efforts to characterize how LRRK2 modifies autophagic activity, the results are controversial or conflicting sometimes in similar models. This fact highlights the huge troubles faced in obtaining a conclusion about the molecular mechanism that links LRRK2 to autophagy in PD, therefore this topic remains under discussion [136,137,138].

## 5. Aggresomes and Role of LRRK2 in Spreading α-Synuclein Toxicity

The aggregation of proteins is a phenomenon that usually happens under physiological conditions during ageing, but in some pathological situations such as PD, its rate accelerates and becomes a key feature of the disease [139]. Aggresomes are cytoplasmatic insoluble complexes emerged from hydrophobic interactions among defective proteins. They are considered a type of extra lysosomal waste and initially they are small protein aggregates that evolve to large cytosolic inclusions [140]. Although aggresomes were initially considered a protective neuronal strategy to isolate harmful proteins and keep potentially neurotoxic proteins well localized in hydrophobic structures, progressive protein accumulation in these insoluble deposits can become detrimental for neurons and trigger neurodegeneration [141,142].

More than 20 years ago, the main component of the classical Lewy’s Bodies and Lewy’s Neurites, the protein α-syn [143,144]—more precisely, the phosphorylated form at serine 129 (pS129) [145]—was characterized. Intraneuronal inclusions in Parkinson’s patients are complex structures that also contain LRRK2 [146,147,148] and a large number of LRRK2 interacting proteins such as the chaperones Hsc70 and Hsp73 [149]. Protein deposition in cytoplasmatic inclusions is dangerous for neurons for two reasons: on the one hand, the physiological protein function is lost when the protein is trapped in aggregates and, on the other hand, individual protein toxicity can be expanded to other neuronal cells in a prion-like manner [150,151,152]. Considering this hypothesis, some LRRK2 variants could potentiate protein transfer to surrounding cells [153]. There is some evidence that corroborates LRRK2 involvement in α-syn aggregation and spread (Figure 2). Interestingly, LRRK2 regulates the clearance of extracellular α-syn aggregates. G2019S LRRK2 mutated astrocytes showed a reduced ability to trap and eliminate α-syn compared to LRRK2 wild-type [154]. LRRK2 and pS129 α-syn co-localize in PD brain samples and LRRK2 promotes its aggregation in some cellular models [155]. Overexpression of LRRK2 wild-type and G2019S mutant induces the aggregation of A53T α-syn variant [156], and the LRRK2 G2019S variant enhanced abnormal α-syn aggregation in Lewy’s Bodies in Parkinson’s patients [147], in human-induced pluripotent stem cell-derived (iPSC) neurons, and PD mouse models [157,158,159,160,161,162], confirming the involvement of LRRK2 itself and the importance of kinase activity of the LRRK2 G2019S mutant in phosphorylation and the progression of α-syn pathology. Moreover, LRRK2 increases α-syn accumulation upon the induction of aggregation with extracellular α-syn preformed fibrils (PFF) in different models. Two different studies in hippocampal neuronal cultures from non-transgenic and transgenic G2019S LRRK2 mice showed that LRRK2 G2019S variant slightly increases the aggregation of pS129 α-syn [158,163]. However, a recent report shows a high increase of pS129 α-syn accumulation in cortical neurons containing a LRRK2 mutant, and LRRK2 knock-out neurons are resistant to pS129 α-syn aggregation induced by PFF [164].

The symptoms and clinical course of familial PD, caused by different LRRK2 point mutations, is similar and indistinguishable from idiopathic cases [165]. Progression of motor symptoms is faster in iPD patients, but some risk-variant carriers such as LRRK2 G2385R progress faster than sporadic cases [166,167]. LRRK2-mutated PD patients also show the classical dopaminergic neuron lost characteristic of PD [168] but, surprisingly, there is no evidence of α-syn aggregation in Lewy’s Bodies in some LRRK2-mutated PD cases [169,170,171,172], with α-syn levels even lower compared to idiopathic PD patients [173]. Instead of typical α-syn aggregates, different proteins are the principal components of protein inclusions, such as accumulated Tau tangles [174,175,176] or TAR DNA-binding protein 43 (TDP-43) [177,178]. In this scenario, LRRK2 promotes neurodegeneration by amplification and spreading pathological proteins such as Tau. The propagation of Tau aggregates, influenced by LRRK2, has been demonstrated in murine models [179]. These pleomorphic neuropathological presentations of protein aggregates hamper the understanding of the role of LRRK2 in PD pathophysiology [180].

## 6. LRRK2 as a Therapeutic Target for PD

To date, there are no disease-modifying therapies (DMT) for PD, but research efforts have tried to define new strategies to stop or delay the progression of the disease [181]. Several of the current anti-Parkinsonian therapeutic strategies include deep brain stimulation (DBS) to minimize motor symptoms and a variety of pharmacological therapies to restore dopamine levels [182]. LRRK2-PD patients are effectively treated with DBS and Levopoda administration, but the beneficial effect of this symptomatic treatment of motor symptoms is temporally limited and it does not modify the clinical course of the disease. Since LRRK2 variants can be considered risk factors for PD, LRRK2 has become an attractive target to devise therapeutic compounds to effectively interfere with PD clinical evolution [183,184,185]. The design of small molecules for controlling LRRK2 kinase activity or the silencing of LRRK2 expression are the best therapeutic options to achieve neuroprotective effects [186].

### 6.1. Pharmacological Strategies: LRRK2 Kinase Inhibitors

**Figure 3:**
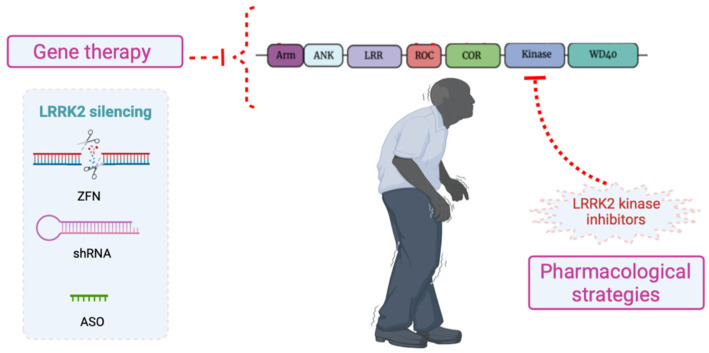
LRRK2 as therapeutic target for PD. Among the therapeutic options to manage LRRK2 activity in PD are included pharmacological strategies that involved the use of LRRK2 kinase inhibitors and gene therapy approaches to knock-down LRRK2 expression by zinc-finger nucleases (ZFN), short-hairpin RNA (shRNA) molecules or antisense oligonucleotides (ASO).

Most PD patients suffering from LRRK2 mutations show an enhanced kinase activity, particularly the G2019S mutation carriers. In those cases, small molecules that act like LRRK2 kinase inhibitors are proposed as an interesting clinical option for controlling PD (Figure 3) [187,188]. However, preclinical studies with LRRK2 kinase inhibitors have shown side effects in peripheral organs such as the kidney or lungs, compromising the safety profile of these compounds [189,190]. Moreover, the inhibition of LRRK2 kinase activity has failed to prevent neuronal damage derived from α-syn spreading [191], which could be related to different mechanisms involved in disease pathogenesis. Apart from these considerations, the LRRK2 kinase inhibitors MLi-2 and PF-066855360 have already been tested in preclinical studies [192], and there are two candidates undergoing clinical trials for PD, DNL201 and DNL151. In 2021, it was announced that DNL151 was included in a last-phase clinical trial with sporadic and LRRK2-PD patients. Although blockade of kinase activity is to date the most explored therapeutic strategy for PD linked to LRRK2 variants, not all LRRK2 mutants show an increase in kinase function limiting studies of substrate phosphorylation. Even in some cases, such as the E193K mutant, LRRK2 kinase activity is not modified. At this point, inhibition of kinase activity, which emerged as a promising therapeutic option, might not be the most appropriate pharmacological choice for all PD cases related to LRRK2 mutations [193] and the development of different strategies seems to be necessary.

### 6.2. Silencing of LRRK2

The concept of gene therapy was defined by Friedman and Roblin in 1972. It is a therapeutic approach for correcting human genetic diseases through the elimination of defective DNA and replacement by corrected exogenous DNA. It involves the substitution or silencing of a specific gene related to the pathological mechanisms of a disease [194].

Loss of function studies of LRRK2 allow the reduction of LRRK2 protein expression levels to minimal amounts and the mimicking of the blockade of protein function. Considering the contribution of LRRK2 to α-syn deposition, targeting LRRK2 is proposed as a potential therapeutic approach to slow the onset and progression of PD derived from α-syn toxicity.

Among the strategies used to modify gene expression in the CNS, the use of viral vectors has been explored [195], in particular adeno-associated virus (AAV) [196,197]. in In addition to LRRK2 knock-out mice, recombinant AAVs (rAVVs) were employed to overexpress α-syn in LRRK2 wild-type and G2019S mutated animal models of PD. Reduction of LRRK2 protein levels in LRRK2 knock-out animals (Figure 3) protected against α-syn toxicity [198], and LRRK2 G2019S mutation worsened the neurodegenerative phenotype in this disease model [199]. Similarly, unlike what happened in LRRK2 wild-type mice, neuronal degeneration increased after overexpression of A53T α-syn using an AAV in LRRK2 G2019S 12-month-old mice [161].

More recently, non-viral gene delivery systems have emerged as good candidates to introduce genetic material into animal models of PD, for targeting disease-modifying genes [200,201]. Several models of synucleinophathy have demonstrated the relationship between LRRK2 and α-syn, and demonstrated the role of LRRK2 aggravating α-syn aggregation and neurotoxicity. In double-transgenic mice models, the co-expression of LRRK2 (wild-type and G2019S) and A53T α-syn acted synergistically to worsen the pathology, but LRRK2 knock-out (by deletion of exon 2 through Cre-LoxP system) reduced α-syn deposition and progression of neuropathological abnormalities [156]. Similar results were found in LRRK2 G2019S and LRRK2 knock-out mice models upon treatment with artificial PFF of α-syn. Absence of LRRK2 decreased α-syn deposition, mainly the pS129 form, compared to LRRK2 mutant [164]. A different strategy was designed to clarify LRRK2 involvement on α-syn aggregation. LRRK2 knock-out, using short-hairpin RNA (shRNA) molecules (Figure 3) in H4 cells, did not alter endogenous α-syn accumulation but, surprisingly, the silencing of LRRK2 in H4 cells co-transfected with α-syn and synphilin-1 enhanced the number of α-syn intracellular inclusions, reduced the size, and did not modify the phosphorylation levels of α-syn [155].

A different approach to block LRRK2 function is based on the reduction of LRRK2 protein levels through treatment with antisense oligonucleotides (ASO) (Figure 3). This methodology allowed the decrease of LRRK2 expression in the nervous system of mice models of PD and the reduction of α-syn aggregation and dopaminergic neuronal damage without modifying LRRK2 expression in other peripheral tissues, such as the kidney and lung, avoiding side effects [202]. The strategy is already in phase I of clinical trials in the REASON study (NCT03976349), which is focused on the characterization of safety, tolerability, and pharmacokinetic profile of the ASO BIIB094 administered to PD patients.

However, some studies have demonstrated α-syn toxic propagation regardless of LRRK2 regulation. The human LRRK2 G2019S variant, as well as the suppression of the LRRK2 wild-type in transgenic mouse models of PD, did not aggravate the behavioral problems or neurochemical phenotype derived from human A53T α-syn expression [203]. In a similar study using double-transgenic mice for α-syn (wild-type and A53T) and LRRK2 (wild-type and G2019S), the LRRK2 G2019S variant did not aggravate α-syn pathology and motor symptoms compared to A53T α-syn phenotype [204]. These findings create doubts about the protective role of LRRK2 suppression in α-syn propagation [205].

## 7. Conclusions

PD is a progressive and chronic neurodegenerative disorder linked to ageing, histologically characterized by damage to and degeneration of dopaminergic neurons and accumulation of protein inclusions, mainly α-syn. The incidence of PD will increase in the coming years due to the ageing of population, and it will become an economic and social problem due to the lack of resolutive clinical therapies. Among the causes that explain PD development and progression, loss of protein homeostasis is one of the main pathological mechanisms. Since the discovery of LRRK2 mutants as one genetic cause of PD, LRRK2 has gained attention when studying the pathological course of the disease, as well as the cellular mechanisms functioning to maintain LRRK2 homeostasis. Among the control pathways responsible for the regulation of LRRK2 equilibrium, several molecular chaperones and the classical clearing mechanism, such as the UPS and ALP, have been described. The imbalance in LRRK2 homeostasis, linked to several point mutations, also facilitates the accumulation and spread of toxic aggregates of α-syn. Although research efforts have demonstrated the relevant role of LRRK2 in PD pathology, new therapeutic options to guarantee LRRK2 homeostasis should be addressed.

## Data Availability

Not applicable.

## References

[1] Kalia L.V., Lang A.E. (2015). Parkinson’s disease. Lancet.

[2] Obeso J.A., Stamelou M., Goetz C.G., Poewe W., Lang A.E., Weintraub D., Burn D., Halliday G.M., Bezard E., Przedborski S. (2017). Past, present, and future of Parkinson’s disease: A special essay on the 200th Anniversary of the Shaking Palsy. Mov. Disord..

[3] Dorsey E.R., Sherer T., Okun M.S., Bloem B.R. (2018). The Emerging Evidence of the Parkinson Pandemic. J. Parkinsons Dis..

[4] Sveinbjornsdottir S. (2016). The clinical symptoms of Parkinson’s disease. J. Neurochem..

[5] Vazquez-Velez G.E., Zoghbi H.Y. (2021). Parkinson’s Disease Genetics and Pathophysiology. Annu. Rev. Neurosci..

[6] Pringsheim T., Jette N., Frolkis A., Steeves T.D. (2014). The prevalence of Parkinson’s disease: A systematic review and meta-analysis. Mov. Disord..

[7] Tosserams A., Araujo R., Pringsheim T., Post B., Darweesh S.K.L., IntHout J., Bloem B.R. (2018). Underrepresentation of women in Parkinson’s disease trials. Mov. Disord..

[8] Deuschl G., Beghi E., Fazekas F., Varga T., Christoforidi K.A., Sipido E., Bassetti C.L., Vos T., Feigin V.L. (2020). The burden of neurological diseases in Europe: An analysis for the Global Burden of Disease Study 2017. Lancet Public Health.

[9] Bjornestad A., Forsaa E.B., Pedersen K.F., Tysnes O.B., Larsen J.P., Alves G. (2016). Risk and course of motor complications in a population-based incident Parkinson’s disease cohort. Parkinsonism Relat. Disord..

[10] Picillo M., Palladino R., Moccia M., Erro R., Amboni M., Vitale C., Barone P., Pellecchia M.T. (2016). Gender and non motor fluctuations in Parkinson’s disease: A prospective study. Parkinsonism Relat. Disord..

[11] Nicoletti A., Vasta R., Mostile G., Nicoletti G., Arabia G., Iliceto G., Lamberti P., Marconi R., Morgante L., Barone P. (2017). Gender effect on non-motor symptoms in Parkinson’s disease: Are men more at risk?. Parkinsonism Relat. Disord..

[12] Fullard M.E., Thibault D.P., Hill A., Fox J., Bhatti D.E., Burack M.A., Dahodwala N., Haberfeld E., Kern D.S., Klepitskava O.S. (2017). Utilization of rehabilitation therapy services in Parkinson disease in the United States. Neurology.

[13] Hall A., Bandres-Ciga S., Diez-Fairen M., Quinn J.P., Billingsley K.J. (2020). Genetic Risk Profiling in Parkinson’s Disease and Utilizing Genetics to Gain Insight into Disease-Related Biological Pathways. Int. J. Mol. Sci..

[14] Chittoor-Vinod V.G., Nichols R.J., Schule B. (2021). Genetic and Environmental Factors Influence the Pleomorphy of LRRK2 Parkinsonism. Int. J. Mol. Sci..

[15] Bandres-Ciga S., Diez-Fairen M., Kim J.J., Singleton A.B. (2020). Genetics of Parkinson’s disease: An introspection of its journey towards precision medicine. Neurobiol. Dis..

[16] Panicker N., Ge P., Dawson V.L., Dawson T.M. (2021). The cell biology of Parkinson’s disease. J. Cell Biol..

[17] Zimprich A., Biskup S., Leitner P., Lichtner P., Farrer M., Lincoln S., Kachergus J., Hulihan M., Uitti R.J., Calne D.B. (2004). Mutations in LRRK2 cause autosomal-dominant parkinsonism with pleomorphic pathology. Neuron.

[18] Brice A. (2005). Genetics of Parkinson’s disease: LRRK2 on the rise. Brain.

[19] Mata I.F., Checkoway H., Hutter C.M., Samii A., Roberts J.W., Kim H.M., Agarwal P., Alvarez V., Ribacoba R., Pastor P. (2012). Common variation in the LRRK2 gene is a risk factor for Parkinson’s disease. Mov. Disord..

[20] Rocha E.M., Keeney M.T., Di Maio R., De Miranda B.R., Greenamyre J.T. (2022). LRRK2 and idiopathic Parkinson’s disease. Trends Neurosci..

[21] Islam M.S., Moore D.J. (2017). Mechanisms of LRRK2-dependent neurodegeneration: Role of enzymatic activity and protein aggregation. Biochem. Soc. Trans..

[22] Bourdenx M., Koulakiotis N.S., Sanoudou D., Bezard E., Dehay B., Tsarbopoulos A. (2017). Protein aggregation and neurodegeneration in prototypical neurodegenerative diseases: Examples of amyloidopathies, tauopathies and synucleinopathies. Prog. Neurobiol..

[23] Mata I.F., Wedemeyer W.J., Farrer M.J., Taylor J.P., Gallo K.A. (2006). LRRK2 in Parkinson’s disease: Protein domains and functional insights. Trends Neurosci..

[24] Gillardon F. (2009). Leucine-rich repeat kinase 2 phosphorylates brain tubulin-beta isoforms and modulates microtubule stability—A point of convergence in parkinsonian neurodegeneration?. J. Neurochem..

[25] Matta S., Van Kolen K., da Cunha R., van den Bogaart G., Mandemakers W., Miskiewicz K., De Bock P.J., Morais V.A., Vilain S., Haddad D. (2012). LRRK2 controls an EndoA phosphorylation cycle in synaptic endocytosis. Neuron.

[26] Marte A., Russo I., Rebosio C., Valente P., Belluzzi E., Pischedda F., Montani C., Lavarello C., Petretto A., Fedele E. (2019). Leucine-rich repeat kinase 2 phosphorylation on synapsin I regulates glutamate release at pre-synaptic sites. J. Neurochem..

[27] Belluzzi E., Gonnelli A., Cirnaru M.D., Marte A., Plotegher N., Russo I., Civiero L., Cogo S., Carrion M.P., Franchin C. (2016). LRRK2 phosphorylates pre-synaptic N-ethylmaleimide sensitive fusion (NSF) protein enhancing its ATPase activity and SNARE complex disassembling rate. Mol. Neurodegener..

[28] Steger M., Tonelli F., Ito G., Davies P., Trost M., Vetter M., Wachter S., Lorentzen E., Duddy G., Wilson S. (2016). Phosphoproteomics reveals that Parkinson’s disease kinase LRRK2 regulates a subset of Rab GTPases. elife.

[29] Sheng Z., Zhang S., Bustos D., Kleinheinz T., Le Pichon C.E., Dominguez S.L., Solanoy H.O., Drummond J., Zhang X., Ding X. (2012). Ser1292 autophosphorylation is an indicator of LRRK2 kinase activity and contributes to the cellular effects of PD mutations. Sci. Transl. Med..

[30] Kamikawaji S., Ito G., Iwatsubo T. (2009). Identification of the autophosphorylation sites of LRRK2. Biochemistry.

[31] Gloeckner C.J., Boldt K., von Zweydorf F., Helm S., Wiesent L., Sarioglu H., Ueffing M. (2010). Phosphopeptide analysis reveals two discrete clusters of phosphorylation in the N-terminus and the Roc domain of the Parkinson-disease associated protein kinase LRRK2. J. Proteome Res..

[32] Marchand A., Drouyer M., Sarchione A., Chartier-Harlin M.C., Taymans J.M. (2020). LRRK2 Phosphorylation, More Than an Epiphenomenon. Front. Neurosci..

[33] Biosa A., Trancikova A., Civiero L., Glauser L., Bubacco L., Greggio E., Moore D.J. (2013). GTPase activity regulates kinase activity and cellular phenotypes of Parkinson’s disease-associated LRRK2. Hum. Mol. Genet..

[34] Ito G., Okai T., Fujino G., Takeda K., Ichijo H., Katada T., Iwatsubo T. (2007). GTP binding is essential to the protein kinase activity of LRRK2, a causative gene product for familial Parkinson’s disease. Biochemistry.

[35] Piccoli G., Onofri F., Cirnaru M.D., Kaiser C.J., Jagtap P., Kastenmuller A., Pischedda F., Marte A., von Zweydorf F., Vogt A. (2014). Leucine-rich repeat kinase 2 binds to neuronal vesicles through protein interactions mediated by its C-terminal WD40 domain. Mol. Cell. Biol..

[36] Porras P., Duesbury M., Fabregat A., Ueffing M., Orchard S., Gloeckner C.J., Hermjakob H. (2015). A visual review of the interactome of LRRK2: Using deep-curated molecular interaction data to represent biology. Proteomics.

[37] Harvey K., Outeiro T.F. (2019). The role of LRRK2 in cell signalling. Biochem. Soc. Trans..

[38] Greggio E., Zambrano I., Kaganovich A., Beilina A., Taymans J.M., Daniels V., Lewis P., Jain S., Ding J., Syed A. (2008). The Parkinson disease-associated leucine-rich repeat kinase 2 (LRRK2) is a dimer that undergoes intramolecular autophosphorylation. J. Biol. Chem..

[39] Guaitoli G., Raimondi F., Gilsbach B.K., Gomez-Llorente Y., Deyaert E., Renzi F., Li X., Schaffner A., Jagtap P.K., Boldt K. (2016). Structural model of the dimeric Parkinson’s protein LRRK2 reveals a compact architecture involving distant interdomain contacts. Proc. Natl. Acad. Sci. USA.

[40] Berger Z., Smith K.A., Lavoie M.J. (2010). Membrane localization of LRRK2 is associated with increased formation of the highly active LRRK2 dimer and changes in its phosphorylation. Biochemistry.

[41] Usmani A., Shavarebi F., Hiniker A. (2021). The Cell Biology of LRRK2 in Parkinson’s Disease. Mol. Cell. Biol..

[42] Piccoli G., Condliffe S.B., Bauer M., Giesert F., Boldt K., De Astis S., Meixner A., Sarioglu H., Vogt-Weisenhorn D.M., Wurst W. (2011). LRRK2 controls synaptic vesicle storage and mobilization within the recycling pool. J. Neurosci..

[43] Biskup S., Moore D.J., Celsi F., Higashi S., West A.B., Andrabi S.A., Kurkinen K., Yu S.W., Savitt J.M., Waldvogel H.J. (2006). Localization of LRRK2 to membranous and vesicular structures in mammalian brain. Ann. Neurol..

[44] Meixner A., Boldt K., Van Troys M., Askenazi M., Gloeckner C.J., Bauer M., Marto J.A., Ampe C., Kinkl N., Ueffing M. (2011). A QUICK screen for Lrrk2 interaction partners--leucine-rich repeat kinase 2 is involved in actin cytoskeleton dynamics. Mol. Cell. Proteom..

[45] Roosen D.A., Cookson M.R. (2016). LRRK2 at the interface of autophagosomes, endosomes and lysosomes. Mol. Neurodegener..

[46] Erb M.L., Moore D.J. (2020). LRRK2 and the Endolysosomal System in Parkinson’s Disease. J. Parkinsons Dis..

[47] Toyofuku T., Okamoto Y., Ishikawa T., Sasawatari S., Kumanogoh A. (2020). LRRK2 regulates endoplasmic reticulum-mitochondrial tethering through the PERK-mediated ubiquitination pathway. EMBO J..

[48] Price A., Manzoni C., Cookson M.R., Lewis P.A. (2018). The LRRK2 signalling system. Cell Tissue Res..

[49] Chen M.L., Wu R.M. (2018). LRRK 2 gene mutations in the pathophysiology of the ROCO domain and therapeutic targets for Parkinson’s disease: A review. J. Biomed. Sci..

[50] Gilks W.P., Abou-Sleiman P.M., Gandhi S., Jain S., Singleton A., Lees A.J., Shaw K., Bhatia K.P., Bonifati V., Quinn N.P. (2005). A common LRRK2 mutation in idiopathic Parkinson’s disease. Lancet.

[51] Benamer H.T., de Silva R. (2010). LRRK2 G2019S in the North African population: A review. Eur. Neurol..

[52] Kachergus J., Mata I.F., Hulihan M., Taylor J.P., Lincoln S., Aasly J., Gibson J.M., Ross O.A., Lynch T., Wiley J. (2005). Identification of a novel LRRK2 mutation linked to autosomal dominant parkinsonism: Evidence of a common founder across European populations. Am. J. Hum. Genet..

[53] Gatto E.M., Parisi V., Converso D.P., Poderoso J.J., Carreras M.C., Marti-Masso J.F., Paisan-Ruiz C. (2013). The LRRK2 G2019S mutation in a series of Argentinean patients with Parkinson’s disease: Clinical and demographic characteristics. Neurosci. Lett..

[54] San Luciano M., Wang C., Ortega R.A., Giladi N., Marder K., Bressman S., Saunders-Pullman R., Michael J.F.F.L.C. (2017). Sex differences in LRRK2 G2019S and idiopathic Parkinson’s Disease. Ann. Clin. Transl. Neurol..

[55] Chen W., Yan X., Lv H., Liu Y., He Z., Luo X. (2020). Gender differences in prevalence of LRRK2-associated Parkinson disease: A meta-analysis of observational studies. Neurosci. Lett..

[56] Tsika E., Nguyen A.P., Dusonchet J., Colin P., Schneider B.L., Moore D.J. (2015). Adenoviral-mediated expression of G2019S LRRK2 induces striatal pathology in a kinase-dependent manner in a rat model of Parkinson’s disease. Neurobiol. Dis..

[57] West A.B., Moore D.J., Choi C., Andrabi S.A., Li X., Dikeman D., Biskup S., Zhang Z., Lim K.L., Dawson V.L. (2007). Parkinson’s disease-associated mutations in LRRK2 link enhanced GTP-binding and kinase activities to neuronal toxicity. Hum. Mol. Genet..

[58] Greggio E. (2012). Role of LRRK2 kinase activity in the pathogenesis of Parkinson’s disease. Biochem. Soc. Trans..

[59] Smith W.W., Pei Z., Jiang H., Dawson V.L., Dawson T.M., Ross C.A. (2006). Kinase activity of mutant LRRK2 mediates neuronal toxicity. Nat. Neurosci..

[60] Schmidt S.H., Knape M.J., Boassa D., Mumdey N., Kornev A.P., Ellisman M.H., Taylor S.S., Herberg F.W. (2019). The dynamic switch mechanism that leads to activation of LRRK2 is embedded in the DFGpsi motif in the kinase domain. Proc. Natl. Acad. Sci. USA.

[61] Paisan-Ruiz C., Lewis P.A., Singleton A.B. (2013). LRRK2: Cause, risk, and mechanism. J. Parkinsons Dis..

[62] Martin I., Kim J.W., Dawson V.L., Dawson T.M. (2014). LRRK2 pathobiology in Parkinson’s disease. J. Neurochem..

[63] Li Y., Dunn L., Greggio E., Krumm B., Jackson G.S., Cookson M.R., Lewis P.A., Deng J. (2009). The R1441C mutation alters the folding properties of the ROC domain of LRRK2. Biochim. Biophys. Acta.

[64] Ohta E., Katayama Y., Kawakami F., Yamamoto M., Tajima K., Maekawa T., Iida N., Hattori S., Obata F. (2009). I(2020)T leucine-rich repeat kinase 2, the causative mutant molecule of familial Parkinson’s disease, has a higher intracellular degradation rate than the wild-type molecule. Biochem. Biophys. Res. Commun..

[65] Greggio E., Jain S., Kingsbury A., Bandopadhyay R., Lewis P., Kaganovich A., van der Brug M.P., Beilina A., Blackinton J., Thomas K.J. (2006). Kinase activity is required for the toxic effects of mutant LRRK2/dardarin. Neurobiol. Dis..

[66] Farrer M.J., Stone J.T., Lin C.H., Dachsel J.C., Hulihan M.M., Haugarvoll K., Ross O.A., Wu R.M. (2007). Lrrk2 G2385R is an ancestral risk factor for Parkinson’s disease in Asia. Parkinsonism Relat. Disord..

[67] Tan E.K., Zhao Y., Skipper L., Tan M.G., Di Fonzo A., Sun L., Fook-Chong S., Tang S., Chua E., Yuen Y. (2007). The LRRK2 Gly2385Arg variant is associated with Parkinson’s disease: Genetic and functional evidence. Hum. Genet..

[68] Cui S.S., Fu R., Du J.J., Lin Y.Q., Huang P., Gao C., Zhou H.Y., Chen S.D. (2021). Sex effects on clinical features in LRRK2 G2385R carriers and non-carriers in Parkinson’s disease. BMC Neurosci..

[69] Zhang P., Fan Y., Ru H., Wang L., Magupalli V.G., Taylor S.S., Alessi D.R., Wu H. (2019). Crystal structure of the WD40 domain dimer of LRRK2. Proc. Natl. Acad. Sci. USA.

[70] Rudenko I.N., Kaganovich A., Hauser D.N., Beylina A., Chia R., Ding J., Maric D., Jaffe H., Cookson M.R. (2012). The G2385R variant of leucine-rich repeat kinase 2 associated with Parkinson’s disease is a partial loss-of-function mutation. Biochem. J..

[71] Carrion M.D.P., Marsicano S., Daniele F., Marte A., Pischedda F., Di Cairano E., Piovesana E., von Zweydorf F., Kremmer E., Gloeckner C.J. (2017). The LRRK2 G2385R variant is a partial loss-of-function mutation that affects synaptic vesicle trafficking through altered protein interactions. Sci. Rep..

[72] Langston R.G., Rudenko I.N., Kumaran R., Hauser D.N., Kaganovich A., Ponce L.B., Mamais A., Ndukwe K., Dillman A.A., Al-Saif A.M. (2019). Differences in Stability, Activity and Mutation Effects Between Human and Mouse Leucine-Rich Repeat Kinase 2. Neurochem. Res..

[73] Pandey N., Fahey M.T., Jong Y.J., O’Malley K.L. (2012). Sequences located within the N-terminus of the PD-linked LRRK2 lead to increased aggregation and attenuation of 6-hydroxydopamine-induced cell death. PLoS ONE.

[74] Perez Carrion M., Pischedda F., Biosa A., Russo I., Straniero L., Civiero L., Guida M., Gloeckner C.J., Ticozzi N., Tiloca C. (2018). The LRRK2 Variant E193K Prevents Mitochondrial Fission Upon MPP+ Treatment by Altering LRRK2 Binding to DRP1. Front. Mol. Neurosci..

[75] Cuanalo-Contreras K., Mukherjee A., Soto C. (2013). Role of protein misfolding and proteostasis deficiency in protein misfolding diseases and aging. Int. J. Cell Biol..

[76] Joshi N., Raveendran A., Nagotu S. (2020). Chaperones and Proteostasis: Role in Parkinson’s Disease. Diseases.

[77] Hu S., Tan J., Qin L., Lv L., Yan W., Zhang H., Tang B., Wang C. (2021). Molecular chaperones and Parkinson’s disease. Neurobiol. Dis..

[78] Limanaqi F., Biagioni F., Gambardella S., Familiari P., Frati A., Fornai F. (2020). Promiscuous Roles of Autophagy and Proteasome in Neurodegenerative Proteinopathies. Int. J. Mol. Sci..

[79] Dikic I. (2017). Proteasomal and Autophagic Degradation Systems. Annu. Rev. Biochem..

[80] Ciechanover A., Kwon Y.T. (2017). Protein Quality Control by Molecular Chaperones in Neurodegeneration. Front. Neurosci..

[81] Voisine C., Pedersen J.S., Morimoto R.I. (2010). Chaperone networks: Tipping the balance in protein folding diseases. Neurobiol. Dis..

[82] Hartl F.U., Bracher A., Hayer-Hartl M. (2011). Molecular chaperones in protein folding and proteostasis. Nature.

[83] Kastle M., Grune T. (2012). Interactions of the proteasomal system with chaperones: Protein triage and protein quality control. Prog. Mol. Biol. Transl. Sci..

[84] Kim Y.E., Hipp M.S., Bracher A., Hayer-Hartl M., Hartl F.U. (2013). Molecular chaperone functions in protein folding and proteostasis. Annu. Rev. Biochem..

[85] Parsell D.A., Kowal A.S., Singer M.A., Lindquist S. (1994). Protein disaggregation mediated by heat-shock protein Hsp104. Nature.

[86] Shorter J. (2011). The mammalian disaggregase machinery: Hsp110 synergizes with Hsp70 and Hsp40 to catalyze protein disaggregation and reactivation in a cell-free system. PLoS ONE.

[87] DeSantis M.E., Leung E.H., Sweeny E.A., Jackrel M.E., Cushman-Nick M., Neuhaus-Follini A., Vashist S., Sochor M.A., Knight M.N., Shorter J. (2012). Operational plasticity enables hsp104 to disaggregate diverse amyloid and nonamyloid clients. Cell.

[88] Doyle S.M., Genest O., Wickner S. (2013). Protein rescue from aggregates by powerful molecular chaperone machines. Nat. Rev. Mol. Cell. Biol..

[89] Hershko A., Ciechanover A. (1992). The ubiquitin system for protein degradation. Annu. Rev. Biochem..

[90] Fernandez-Fernandez M.R., Gragera M., Ochoa-Ibarrola L., Quintana-Gallardo L., Valpuesta J.M. (2017). Hsp70—A master regulator in protein degradation. FEBS Lett..

[91] Chondrogianni N., Gonos E.S. (2012). Structure and function of the ubiquitin-proteasome system: Modulation of components. Prog. Mol. Biol. Transl. Sci..

[92] De Poot S.A.H., Tian G., Finley D. (2017). Meddling with Fate: The Proteasomal Deubiquitinating Enzymes. J. Mol. Biol..

[93] Lichtenberg M., Mansilla A., Zecchini V.R., Fleming A., Rubinsztein D.C. (2011). The Parkinson’s disease protein LRRK2 impairs proteasome substrate clearance without affecting proteasome catalytic activity. Cell Death Dis..

[94] Ko H.S., Bailey R., Smith W.W., Liu Z., Shin J.H., Lee Y.I., Zhang Y.J., Jiang H., Ross C.A., Moore D.J. (2009). CHIP regulates leucine-rich repeat kinase-2 ubiquitination, degradation, and toxicity. Proc. Natl. Acad. Sci. USA.

[95] Ding X., Goldberg M.S. (2009). Regulation of LRRK2 stability by the E3 ubiquitin ligase CHIP. PLoS ONE.

[96] Rudenko I.N., Kaganovich A., Langston R.G., Beilina A., Ndukwe K., Kumaran R., Dillman A.A., Chia R., Cookson M.R. (2017). The G2385R risk factor for Parkinson’s disease enhances CHIP-dependent intracellular degradation of LRRK2. Biochem. J..

[97] Dachsel J.C., Taylor J.P., Mok S.S., Ross O.A., Hinkle K.M., Bailey R.M., Hines J.H., Szutu J., Madden B., Petrucelli L. (2007). Identification of potential protein interactors of Lrrk2. Parkinsonism Relat. Disord..

[98] Gloeckner C.J., Kinkl N., Schumacher A., Braun R.J., O’Neill E., Meitinger T., Kolch W., Prokisch H., Ueffing M. (2006). The Parkinson disease causing LRRK2 mutation I2020T is associated with increased kinase activity. Hum. Mol. Genet..

[99] Wang L., Xie C., Greggio E., Parisiadou L., Shim H., Sun L., Chandran J., Lin X., Lai C., Yang W.J. (2008). The chaperone activity of heat shock protein 90 is critical for maintaining the stability of leucine-rich repeat kinase 2. J. Neurosci..

[100] Fukuzono T., Pastuhov S.I., Fukushima O., Li C., Hattori A., Iemura S., Natsume T., Shibuya H., Hanafusa H., Matsumoto K. (2016). Chaperone complex BAG2-HSC70 regulates localization of Caenorhabditis elegans leucine-rich repeat kinase LRK-1 to the Golgi. Genes Cells.

[101] Bingol B. (2018). Autophagy and lysosomal pathways in nervous system disorders. Mol. Cell Neurosci..

[102] Menzies F.M., Fleming A., Caricasole A., Bento C.F., Andrews S.P., Ashkenazi A., Fullgrabe J., Jackson A., Jimenez Sanchez M., Karabiyik C. (2017). Autophagy and Neurodegeneration: Pathogenic Mechanisms and Therapeutic Opportunities. Neuron.

[103] Cuervo A.M., Bergamini E., Brunk U.T., Droge W., Ffrench M., Terman A. (2005). Autophagy and aging: The importance of maintaining “clean” cells. Autophagy.

[104] Sarkar S., Ravikumar B., Rubinsztein D.C. (2009). Autophagic clearance of aggregate-prone proteins associated with neurodegeneration. Methods Enzymol..

[105] Metcalf D.J., Garcia-Arencibia M., Hochfeld W.E., Rubinsztein D.C. (2012). Autophagy and misfolded proteins in neurodegeneration. Exp. Neurol..

[106] Cuervo A.M. (2008). Autophagy and aging: Keeping that old broom working. Trends Genet..

[107] Johnson M.E., Stecher B., Labrie V., Brundin L., Brundin P. (2019). Triggers, Facilitators, and Aggravators: Redefining Parkinson’s Disease Pathogenesis. Trends Neurosci..

[108] Moors T.E., Hoozemans J.J., Ingrassia A., Beccari T., Parnetti L., Chartier-Harlin M.C., van de Berg W.D. (2017). Therapeutic potential of autophagy-enhancing agents in Parkinson’s disease. Mol. Neurodegener..

[109] Finkbeiner S. (2020). The Autophagy Lysosomal Pathway and Neurodegeneration. Cold Spring Harb. Perspect. Biol..

[110] Li W.W., Li J., Bao J.K. (2012). Microautophagy: Lesser-known self-eating. Cell. Mol. Life Sci..

[111] Olsvik H.L., Svenning S., Abudu Y.P., Brech A., Stenmark H., Johansen T., Mejlvang J. (2019). Endosomal microautophagy is an integrated part of the autophagic response to amino acid starvation. Autophagy.

[112] Dice J.F. (1990). Peptide sequences that target cytosolic proteins for lysosomal proteolysis. Trends Biochem. Sci..

[113] Cuervo A.M., Dice J.F. (1996). A receptor for the selective uptake and degradation of proteins by lysosomes. Science.

[114] Kaushik S., Cuervo A.M. (2012). Chaperone-mediated autophagy: A unique way to enter the lysosome world. Trends Cell Biol..

[115] Sala G., Marinig D., Arosio A., Ferrarese C. (2016). Role of Chaperone-Mediated Autophagy Dysfunctions in the Pathogenesis of Parkinson’s Disease. Front. Mol. Neurosci..

[116] Massey A.C., Zhang C., Cuervo A.M. (2006). Chaperone-mediated autophagy in aging and disease. Curr. Top. Dev. Biol..

[117] Orenstein S.J., Kuo S.H., Tasset I., Arias E., Koga H., Fernandez-Carasa I., Cortes E., Honig L.S., Dauer W., Consiglio A. (2013). Interplay of LRRK2 with chaperone-mediated autophagy. Nat. Neurosci..

[118] Ho P.W., Leung C.T., Liu H., Pang S.Y., Lam C.S., Xian J., Li L., Kung M.H., Ramsden D.B., Ho S.L. (2020). Age-dependent accumulation of oligomeric SNCA/alpha-synuclein from impaired degradation in mutant LRRK2 knockin mouse model of Parkinson disease: Role for therapeutic activation of chaperone-mediated autophagy (CMA). Autophagy.

[119] Klionsky D.J. (2005). Autophagy. Curr. Biol..

[120] Codogno P., Mehrpour M., Proikas-Cezanne T. (2011). Canonical and non-canonical autophagy: Variations on a common theme of self-eating?. Nat. Rev. Mol. Cell. Biol..

[121] Pickles S., Vigie P., Youle R.J. (2018). Mitophagy and Quality Control Mechanisms in Mitochondrial Maintenance. Curr. Biol..

[122] Manjithaya R., Nazarko T.Y., Farre J.C., Subramani S. (2010). Molecular mechanism and physiological role of pexophagy. FEBS Lett..

[123] Gatica D., Lahiri V., Klionsky D.J. (2018). Cargo recognition and degradation by selective autophagy. Nat. Cell Biol..

[124] Karabiyik C., Lee M.J., Rubinsztein D.C. (2017). Autophagy impairment in Parkinson’s disease. Essays Biochem..

[125] Plowey E.D., Cherra S.J., Liu Y.J., Chu C.T. (2008). Role of autophagy in G2019S-LRRK2-associated neurite shortening in differentiated SH-SY5Y cells. J. Neurochem..

[126] Alegre-Abarrategui J., Christian H., Lufino M.M., Mutihac R., Venda L.L., Ansorge O., Wade-Martins R. (2009). LRRK2 regulates autophagic activity and localizes to specific membrane microdomains in a novel human genomic reporter cellular model. Hum. Mol. Genet..

[127] Gomez-Suaga P., Luzon-Toro B., Churamani D., Zhang L., Bloor-Young D., Patel S., Woodman P.G., Churchill G.C., Hilfiker S. (2012). Leucine-rich repeat kinase 2 regulates autophagy through a calcium-dependent pathway involving NAADP. Hum. Mol. Genet..

[128] Bravo-San Pedro J.M., Niso-Santano M., Gomez-Sanchez R., Pizarro-Estrella E., Aiastui-Pujana A., Gorostidi A., Climent V., Lopez de Maturana R., Sanchez-Pernaute R., Lopez de Munain A. (2013). The LRRK2 G2019S mutant exacerbates basal autophagy through activation of the MEK/ERK pathway. Cell. Mol. Life Sci..

[129] Manzoni C., Mamais A., Dihanich S., Abeti R., Soutar M.P.M., Plun-Favreau H., Giunti P., Tooze S.A., Bandopadhyay R., Lewis P.A. (2013). Inhibition of LRRK2 kinase activity stimulates macroautophagy. Biochim. Biophys. Acta.

[130] Manzoni C., Mamais A., Dihanich S., McGoldrick P., Devine M.J., Zerle J., Kara E., Taanman J.W., Healy D.G., Marti-Masso J.F. (2013). Pathogenic Parkinson’s disease mutations across the functional domains of LRRK2 alter the autophagic/lysosomal response to starvation. Biochem. Biophys. Res. Commun..

[131] Dodson M.W., Leung L.K., Lone M., Lizzio M.A., Guo M. (2014). Novel ethyl methanesulfonate (EMS)-induced null alleles of the Drosophila homolog of LRRK2 reveal a crucial role in endolysosomal functions and autophagy in vivo. Dis. Models Mech..

[132] Ramonet D., Daher J.P., Lin B.M., Stafa K., Kim J., Banerjee R., Westerlund M., Pletnikova O., Glauser L., Yang L. (2011). Dopaminergic neuronal loss, reduced neurite complexity and autophagic abnormalities in transgenic mice expressing G2019S mutant LRRK2. PLoS ONE.

[133] Tong Y., Yamaguchi H., Giaime E., Boyle S., Kopan R., Kelleher R.J., Shen J. (2010). Loss of leucine-rich repeat kinase 2 causes impairment of protein degradation pathways, accumulation of alpha-synuclein, and apoptotic cell death in aged mice. Proc. Natl. Acad. Sci. USA.

[134] Tong Y., Giaime E., Yamaguchi H., Ichimura T., Liu Y., Si H., Cai H., Bonventre J.V., Shen J. (2012). Loss of leucine-rich repeat kinase 2 causes age-dependent bi-phasic alterations of the autophagy pathway. Mol. Neurodegener..

[135] Mamais A., Manzoni C., Nazish I., Arber C., Sonustun B., Wray S., Warner T.T., Cookson M.R., Lewis P.A., Bandopadhyay R. (2018). Analysis of macroautophagy related proteins in G2019S LRRK2 Parkinson’s disease brains with Lewy body pathology. Brain Res..

[136] Albanese F., Novello S., Morari M. (2019). Autophagy and LRRK2 in the Aging Brain. Front. Neurosci..

[137] Manzoni C., Lewis P.A. (2017). LRRK2 and Autophagy. Adv. Neurobiol..

[138] Madureira M., Connor-Robson N., Wade-Martins R. (2020). “LRRK2: Autophagy and Lysosomal Activity”. Front. Neurosci..

[139] Thal D.R., Del Tredici K., Braak H. (2004). Neurodegeneration in normal brain aging and disease. Sci. Aging Knowl. Environ..

[140] Kopito R.R. (2000). Aggresomes, inclusion bodies and protein aggregation. Trends Cell Biol..

[141] Ross C.A., Poirier M.A. (2005). Opinion: What is the role of protein aggregation in neurodegeneration?. Nat. Rev. Mol. Cell. Biol..

[142] Chartier S., Duyckaerts C. (2018). Is Lewy pathology in the human nervous system chiefly an indicator of neuronal protection or of toxicity?. Cell Tissue Res..

[143] Duda J.E., Lee V.M., Trojanowski J.Q. (2000). Neuropathology of synuclein aggregates. J. Neurosci. Res..

[144] Spillantini M.G., Schmidt M.L., Lee V.M., Trojanowski J.Q., Jakes R., Goedert M. (1997). Alpha-synuclein in Lewy bodies. Nature.

[145] Walker D.G., Lue L.F., Adler C.H., Shill H.A., Caviness J.N., Sabbagh M.N., Akiyama H., Serrano G.E., Sue L.I., Beach T.G. (2013). Changes in properties of serine 129 phosphorylated alpha-synuclein with progression of Lewy-type histopathology in human brains. Exp. Neurol..

[146] Perry G., Zhu X., Babar A.K., Siedlak S.L., Yang Q., Ito G., Iwatsubo T., Smith M.A., Chen S.G. (2008). Leucine-rich repeat kinase 2 colocalizes with alpha-synuclein in Parkinson’s disease, but not tau-containing deposits in tauopathies. Neurodegener. Dis..

[147] Ross O.A., Toft M., Whittle A.J., Johnson J.L., Papapetropoulos S., Mash D.C., Litvan I., Gordon M.F., Wszolek Z.K., Farrer M.J. (2006). Lrrk2 and Lewy body disease. Ann. Neurol..

[148] Alegre-Abarrategui J., Ansorge O., Esiri M., Wade-Martins R. (2008). LRRK2 is a component of granular alpha-synuclein pathology in the brainstem of Parkinson’s disease. Neuropathol. Appl. Neurobiol..

[149] Leverenz J.B., Umar I., Wang Q., Montine T.J., McMillan P.J., Tsuang D.W., Jin J., Pan C., Shin J., Zhu D. (2007). Proteomic identification of novel proteins in cortical lewy bodies. Brain Pathol..

[150] Brundin P., Melki R. (2017). Prying into the Prion Hypothesis for Parkinson’s Disease. J. Neurosci..

[151] Walsh D.M., Selkoe D.J. (2016). A critical appraisal of the pathogenic protein spread hypothesis of neurodegeneration. Nat. Rev. Neurosci..

[152] Jucker M., Walker L.C. (2013). Self-propagation of pathogenic protein aggregates in neurodegenerative diseases. Nature.

[153] Lewis P.A. (2018). Mutations in LRRK2 amplify cell-to-cell protein aggregate propagation: A hypothesis. Acta Neuropathol..

[154] Streubel-Gallasch L., Giusti V., Sandre M., Tessari I., Plotegher N., Giusto E., Masato A., Iovino L., Battisti I., Arrigoni G. (2021). Parkinson’s Disease-Associated LRRK2 Interferes with Astrocyte-Mediated Alpha-Synuclein Clearance. Mol. Neurobiol..

[155] Guerreiro P.S., Huang Y., Gysbers A., Cheng D., Gai W.P., Outeiro T.F., Halliday G.M. (2013). LRRK2 interactions with alpha-synuclein in Parkinson’s disease brains and in cell models. J. Mol. Med..

[156] Lin X., Parisiadou L., Gu X.L., Wang L., Shim H., Sun L., Xie C., Long C.X., Yang W.J., Ding J. (2009). Leucine-rich repeat kinase 2 regulates the progression of neuropathology induced by Parkinson’s-disease-related mutant alpha-synuclein. Neuron.

[157] Bieri G., Brahic M., Bousset L., Couthouis J., Kramer N.J., Ma R., Nakayama L., Monbureau M., Defensor E., Schule B. (2019). LRRK2 modifies alpha-syn pathology and spread in mouse models and human neurons. Acta Neuropathol..

[158] Volpicelli-Daley L.A., Abdelmotilib H., Liu Z., Stoyka L., Daher J.P., Milnerwood A.J., Unni V.K., Hirst W.D., Yue Z., Zhao H.T. (2016). G2019S-LRRK2 Expression Augments alpha-Synuclein Sequestration into Inclusions in Neurons. J. Neurosci..

[159] Longo F., Mercatelli D., Novello S., Arcuri L., Brugnoli A., Vincenzi F., Russo I., Berti G., Mabrouk O.S., Kennedy R.T. (2017). Age-dependent dopamine transporter dysfunction and Serine129 phospho-alpha-synuclein overload in G2019S LRRK2 mice. Acta Neuropathol. Commun..

[160] Xiong Y., Neifert S., Karuppagounder S.S., Stankowski J.N., Lee B.D., Grima J.C., Chen G., Ko H.S., Lee Y., Swing D. (2017). Overexpression of Parkinson’s Disease-Associated Mutation LRRK2 G2019S in Mouse Forebrain Induces Behavioral Deficits and alpha-Synuclein Pathology. eNeuro.

[161] Novello S., Arcuri L., Dovero S., Dutheil N., Shimshek D.R., Bezard E., Morari M. (2018). G2019S LRRK2 mutation facilitates alpha-synuclein neuropathology in aged mice. Neurobiol. Dis..

[162] Schapansky J., Khasnavis S., DeAndrade M.P., Nardozzi J.D., Falkson S.R., Boyd J.D., Sanderson J.B., Bartels T., Melrose H.L., LaVoie M.J. (2018). Familial knockin mutation of LRRK2 causes lysosomal dysfunction and accumulation of endogenous insoluble alpha-synuclein in neurons. Neurobiol. Dis..

[163] Henderson M.X., Peng C., Trojanowski J.Q., Lee V.M.Y. (2018). LRRK2 activity does not dramatically alter alpha-synuclein pathology in primary neurons. Acta Neuropathol. Commun.

[164] MacIsaac S., Quevedo Melo T., Zhang Y., Volta M., Farrer M.J., Milnerwood A.J. (2020). Neuron-autonomous susceptibility to induced synuclein aggregation is exacerbated by endogenous Lrrk2 mutations and ameliorated by Lrrk2 genetic knock-out. Brain Commun..

[165] Marras C., Alcalay R.N., Caspell-Garcia C., Coffey C., Chan P., Duda J.E., Facheris M.F., Fernandez-Santiago R., Ruiz-Martinez J., Mestre T. (2016). Motor and nonmotor heterogeneity of LRRK2-related and idiopathic Parkinson’s disease. Mov. Disord..

[166] Alcalay R.N., Mirelman A., Saunders-Pullman R., Tang M.X., Mejia Santana H., Raymond D., Roos E., Orbe-Reilly M., Gurevich T., Bar Shira A. (2013). Parkinson disease phenotype in Ashkenazi Jews with and without LRRK2 G2019S mutations. Mov. Disord..

[167] Oosterveld L.P., Allen J.C., Ng E.Y., Seah S.H., Tay K.Y., Au W.L., Tan E.K., Tan L.C. (2015). Greater motor progression in patients with Parkinson disease who carry LRRK2 risk variants. Neurology.

[168] Schneider S.A., Alcalay R.N. (2017). Neuropathology of genetic synucleinopathies with parkinsonism: Review of the literature. Mov. Disord..

[169] Marti-Masso J.F., Ruiz-Martinez J., Bolano M.J., Ruiz I., Gorostidi A., Moreno F., Ferrer I., Lopez de Munain A. (2009). Neuropathology of Parkinson’s disease with the R1441G mutation in LRRK2. Mov. Disord..

[170] Santpere G., Ferrer I. (2009). LRRK2 and neurodegeneration. Acta Neuropathol..

[171] Kalia L.V., Lang A.E., Hazrati L.N., Fujioka S., Wszolek Z.K., Dickson D.W., Ross O.A., Van Deerlin V.M., Trojanowski J.Q., Hurtig H.I. (2015). Clinical correlations with Lewy body pathology in LRRK2-related Parkinson disease. JAMA Neurol..

[172] O’Hara D.M., Pawar G., Kalia S.K., Kalia L.V. (2020). LRRK2 and alpha-Synuclein: Distinct or Synergistic Players in Parkinson’s Disease?. Front. Neurosci..

[173] Mamais A., Raja M., Manzoni C., Dihanich S., Lees A., Moore D., Lewis P.A., Bandopadhyay R. (2013). Divergent alpha-synuclein solubility and aggregation properties in G2019S LRRK2 Parkinson’s disease brains with Lewy Body pathology compared to idiopathic cases. Neurobiol. Dis..

[174] Henderson M.X., Sengupta M., Trojanowski J.Q., Lee V.M.Y. (2019). Alzheimer’s disease tau is a prominent pathology in LRRK2 Parkinson’s disease. Acta Neuropathol. Commun..

[175] Rajput A., Dickson D.W., Robinson C.A., Ross O.A., Dachsel J.C., Lincoln S.J., Cobb S.A., Rajput M.L., Farrer M.J. (2006). Parkinsonism, Lrrk2 G2019S, and tau neuropathology. Neurology.

[176] Wszolek Z.K., Pfeiffer R.F., Tsuboi Y., Uitti R.J., McComb R.D., Stoessl A.J., Strongosky A.J., Zimprich A., Muller-Myhsok B., Farrer M.J. (2004). Autosomal dominant parkinsonism associated with variable synuclein and tau pathology. Neurology.

[177] Wider C., Dickson D.W., Wszolek Z.K. (2010). Leucine-rich repeat kinase 2 gene-associated disease: Redefining genotype-phenotype correlation. Neurodegener. Dis..

[178] Ling H., Kara E., Bandopadhyay R., Hardy J., Holton J., Xiromerisiou G., Lees A., Houlden H., Revesz T. (2013). TDP-43 pathology in a patient carrying G2019S LRRK2 mutation and a novel p.Q124E MAPT. Neurobiol. Aging.

[179] Nguyen A.P.T., Daniel G., Valdes P., Islam M.S., Schneider B.L., Moore D.J. (2018). G2019S LRRK2 enhances the neuronal transmission of tau in the mouse brain. Hum. Mol. Genet..

[180] Tolosa E., Garrido A., Scholz S.W., Poewe W. (2021). Challenges in the diagnosis of Parkinson’s disease. Lancet Neurol..

[181] Vijiaratnam N., Simuni T., Bandmann O., Morris H.R., Foltynie T. (2021). Progress towards therapies for disease modification in Parkinson’s disease. Lancet Neurol..

[182] Bloem B.R., Okun M.S., Klein C. (2021). Parkinson’s disease. Lancet.

[183] Chen J., Chen Y., Pu J. (2018). Leucine-Rich Repeat Kinase 2 in Parkinson’s Disease: Updated from Pathogenesis to Potential Therapeutic Target. Eur. Neurol..

[184] Wojewska D.N., Kortholt A. (2021). LRRK2 Targeting Strategies as Potential Treatment of Parkinson’s Disease. Biomolecules.

[185] Senkevich K., Rudakou U., Gan-Or Z. (2022). New therapeutic approaches to Parkinson’s disease targeting GBA, LRRK2 and Parkin. Neuropharmacology.

[186] Tolosa E., Vila M., Klein C., Rascol O. (2020). LRRK2 in Parkinson disease: Challenges of clinical trials. Nat. Rev. Neurol..

[187] West A.B. (2015). Ten years and counting: Moving leucine-rich repeat kinase 2 inhibitors to the clinic. Mov. Disord..

[188] Cogo S., Greggio E., Lewis P.A. (2017). Leucine Rich Repeat Kinase 2: Beyond Parkinson’s and beyond kinase inhibitors. Expert Opin. Ther. Targets.

[189] Fuji R.N., Flagella M., Baca M., Baptista M.A., Brodbeck J., Chan B.K., Fiske B.K., Honigberg L., Jubb A.M., Katavolos P. (2015). Effect of selective LRRK2 kinase inhibition on nonhuman primate lung. Sci. Transl. Med..

[190] Taymans J.M., Greggio E. (2016). LRRK2 Kinase Inhibition as a Therapeutic Strategy for Parkinson’s Disease, Where Do We Stand?. Curr. Neuropharmacol..

[191] Henderson M.X., Sengupta M., McGeary I., Zhang B., Olufemi M.F., Brown H., Trojanowski J.Q., Lee V.M.Y. (2019). LRRK2 inhibition does not impart protection from alpha-synuclein pathology and neuron death in non-transgenic mice. Acta Neuropathol. Commun..

[192] West A.B. (2017). Achieving neuroprotection with LRRK2 kinase inhibitors in Parkinson disease. Exp. Neurol..

[193] Rudenko I.N., Chia R., Cookson M.R. (2012). Is inhibition of kinase activity the only therapeutic strategy for LRRK2-associated Parkinson’s disease?. BMC Med..

[194] Friedmann T., Roblin R. (1972). Gene therapy for human genetic disease?. Science.

[195] Lentz T.B., Gray S.J., Samulski R.J. (2012). Viral vectors for gene delivery to the central nervous system. Neurobiol. Dis..

[196] Macdonald J., Marx J., Buning H. (2021). Capsid-Engineering for Central Nervous System-Directed Gene Therapy with Adeno-Associated Virus Vectors. Hum. Gene Ther..

[197] Fajardo-Serrano A., Rico A.J., Roda E., Honrubia A., Arrieta S., Ariznabarreta G., Chocarro J., Lorenzo-Ramos E., Pejenaute A., Vazquez A. (2021). Adeno-Associated Viral Vectors as Versatile Tools for Parkinson’s Research, Both for Disease Modeling Purposes and for Therapeutic Uses. Int. J. Mol. Sci..

[198] Daher J.P., Volpicelli-Daley L.A., Blackburn J.P., Moehle M.S., West A.B. (2014). Abrogation of alpha-synuclein-mediated dopaminergic neurodegeneration in LRRK2-deficient rats. Proc. Natl. Acad. Sci. USA.

[199] Daher J.P., Abdelmotilib H.A., Hu X., Volpicelli-Daley L.A., Moehle M.S., Fraser K.B., Needle E., Chen Y., Steyn S.J., Galatsis P. (2015). Leucine-rich Repeat Kinase 2 (LRRK2) Pharmacological Inhibition Abates alpha-Synuclein Gene-induced Neurodegeneration. J. Biol. Chem..

[200] Axelsen T.M., Woldbye D.P.D. (2018). Gene Therapy for Parkinson’s Disease, An Update. J. Parkinsons Dis..

[201] Sudhakar V., Richardson R.M. (2018). Gene Therapy for Parkinson’s Disease. Prog. Neurol. Surg..

[202] Zhao H.T., John N., Delic V., Ikeda-Lee K., Kim A., Weihofen A., Swayze E.E., Kordasiewicz H.B., West A.B., Volpicelli-Daley L.A. (2017). LRRK2 Antisense Oligonucleotides Ameliorate alpha-Synuclein Inclusion Formation in a Parkinson’s Disease Mouse Model. Mol. Ther. Nucleic Acids.

[203] Daher J.P., Pletnikova O., Biskup S., Musso A., Gellhaar S., Galter D., Troncoso J.C., Lee M.K., Dawson T.M., Dawson V.L. (2012). Neurodegenerative phenotypes in an A53T alpha-synuclein transgenic mouse model are independent of LRRK2. Hum. Mol. Genet..

[204] Herzig M.C., Bidinosti M., Schweizer T., Hafner T., Stemmelen C., Weiss A., Danner S., Vidotto N., Stauffer D., Barske C. (2012). High LRRK2 levels fail to induce or exacerbate neuronal alpha-synucleinopathy in mouse brain. PLoS ONE.

[205] Cresto N., Gardier C., Gubinelli F., Gaillard M.C., Liot G., West A.B., Brouillet E. (2019). The unlikely partnership between LRRK2 and alpha-synuclein in Parkinson’s disease. Eur. J. Neurosci..

